# Supplementary material for: Predictive value of immunoglobulin G, activated partial thromboplastin time, platelet, and indirect bilirubin for delayed viral clearance in patients infected with the Omicron variant
Source: PeerJ. 2023 May 19;11:e15443. doi: 10.7717/peerj.15443 (PMC10202103; doi:10.7717/peerj.15443)
Supplement: Supplemental Information 2 [file peerj-11-15443-s002.docx]

**Table S1**. Correlation between age, commonly used hematological parameters, and virus shedding duration (Pearson correlation)

| Hematological parameters | Pearson’s correlation coefficient | P value |
| --- | --- | --- |
| Age | 0.123964657 | 0.02909259 |
| Dbil | 0.102873361 | 0.001304526 |
| Tbil | 0.138621731 | 0.0000141407873799927 |
| CRP | 0.143307753 | 0.011535247 |
| IgG | -0.293504393 | 4.23E-08 |
| APTT | 0.13347399 | 5.58E-06 |
| Myoglobin | 0.126966547 | 0.000595263 |
| lymphocyte | -0.14756367510920665 | 7.054779996482462e-08 |
| Platelet | -0.221338742 | 3.91E-16 |
| Leukocyte | -0.137932105 | 4.79E-07 |
| Creatinine | 0.077418835 | 0.006731293 |
| Neutrophil | -0.09751253 | 0.000384355 |
| γ-glutamyltransferase | 0.028131427 | 0.380487128 |
| alanine aminotransferase | -0.002351371 | 0.94157555 |
| aspartate aminotransferase | -0.005945617 | 0.852978077 |
| Alkaline Phosphatase. | 0.023200464 | 0.469538459 |
| Albumin | -0.072032887 | 0.0245694 |
| Globulin | -0.032084056 | 0.317173979260548 |
| Total protein | -0.085885083 | 0.007320534 |
| Interleukin-6 | 0.01666549 | 0.770086947 |
| Procalcitonin | 0.047360151 | 0.405996631 |
| IgM | -0.060338739 | 0.270065909 |
| D-Dimer | 0.06702449 | 0.02302645 |
| fibrin degradation products (FDP) | -0.026612906 | 0.367234424 |
| Fibrinogen | -0.01354262 | 0.646396332 |
| International Normalized Ratio(INR) | 0.057503049 | 0.05123385 |
| Prothrombin Time | 0.057999423 | 0.049255586 |
| thrombin time | 0.043101536 | 0.144088595 |
| Uric acid | -0.025251312 | 0.37741285 |
| Urea | 0.050932002 | 0.074875239 |
| Troponin-I | -0.088196386 | 0.017302344 |
| Creatine kinase-MB isoenzyme | -0.010020623 | 0.787226909 |
| N-Terminal Pro-B-Type Natriuretic Peptide | 0.043722321 | 0.238705322 |
| Eosinophils | -0.089911219 | 0.00106545 |
| Hematocrit | 0.017180176 | 0.532552293 |
| Hemoglobin | -0.005163122 | 0.851228429 |
| Lymphocyte percentage | -0.070396927 | 0.010456983 |
| MCH | -0.047970479 | 0.081241466 |
| MCHC | -0.091432296 | 0.000873951 |
| MCV | -0.00149184 | 0.956782991 |
| Monocyte percentage | 0.102133391 | 0.000199445 |
| Monocyte | -0.032178006 | 0.242336604 |
| Neutrophil percentage | 0.040466313 | 0.141416085 |
| red blood cell | 0.019979311 | 0.467949496 |
| Glucose | -0.012366992 | 0.737834629 |
| Chloride | 0.017742937 | 0.631052631 |
| Potassium | 0.01078885 | 0.770282104 |
| Sodium | -0.03429732 | 0.35313726 |
